# Supplementary material for: Diversity, antibacterial and phytotoxic activities of actinomycetes associated with Periplaneta fuliginosa
Source: PeerJ. 2024 Nov 25;12:e18575. doi: 10.7717/peerj.18575 (PMC11604042; doi:10.7717/peerj.18575)
Supplement: Supplemental Information 1 [file peerj-12-18575-s001.docx]

**Table S1.** Media used for isolation and cultivation in this study.

**Table S2.** Phylogenetic analysis of cultivable actinomycetes associated with *P. fuliginosa*.

**Figure S1.** Colony morphology of part symbiotic actinomycetes.

**Figure S2.** Neighbor-joining phylogenetic tree of 16S rRNA sequences of ZLC-87

**Figure S3.** The inhibitory effect of compound **4** on *E. crusgalli* and *A. theophrasti*.

**Figure S4.** The ^1^H NMR spectrum of compound **1** (600 MHz, CDCl_3_).

**Figure S5.** The ^13^C NMR spectrum of compound **1** (150 MHz, CDCl_3_).

**Figure S6.** The HR-ESI-MS spectrum of compound **1**.

**Figure S7.** The ^1^H NMR spectrum of compound **2** (600 MHz, DMSO- *d_6_*).

**Figure S8.** The ^13^C NMR spectrum of compound **2** (150 MHz, DMSO- *d_6_*).

**Figure S9.** The HR-ESI-MS spectrum of compound **2**.

**Figure S10.** The ^1^H NMR spectrum of compound **3** (600 MHz, CDCl_3_).

**Figure S11.** The ^13^C NMR spectrum of compound **3** (150 MHz, CDCl_3_).

**Figure S12.** The HR-ESI-MS spectrum of compound **3**.

**Figure S13.** The ^1^H NMR spectrum of compound **4** (600 MHz, Acetone-*d_6_*).

**Figure S14.** The ^13^C NMR spectrum of compound **4** (150 MHz, Acetone-*d_6_*).

**Figure S15.** The HR-ESI-MS spectrum of compound **4**.

**Figure S16.** The ^1^H NMR spectrum of compound **5** (600 MHz, CDCl_3_).

**Figure S17.** The ^13^C NMR spectrum of compound **5** (150 MHz, CDCl_3_).

**Figure S18.** The HR-ESI-MS spectrum of compound **5**.

**Figure S19.** The ^1^H NMR spectrum of compound **6** (600 MHz, DMSO- *d_6_*).

**Figure S20.** The ^13^C NMR spectrum of compound **6** (150 MHz, DMSO- *d_6_*).

**Figure S21.** The HR-ESI-MS spectrum of compound **6**.

**Figure S22.** The ^1^H NMR spectrum of compound **7** (600 MHz, Acetone-*d_6_*).

**Figure S23.** The ^13^C NMR spectrum of compound **7** (150 MHz, Acetone-*d_6_*).

**Figure S24.** The HR-ESI-MS spectrum of compound **7**.

**Table S1.** Media used for isolation and cultivation in this study.

| **Usage** | **Media** | **Components** |
| --- | --- | --- |
| Isolation | ISP2 (ISP Medium No. 2) | Malt extract 10.0 g, yeast extract 4.0 g, glucose 4.0 g, agar 18.0 g, H_2_O 1000 mL, pH 7.0 |
|  | CA (chitin agar medium) | Chitin 4.0 g, K_2_HPO_4_·3H_2_O 0.7 g, KH_2_PO_4_ 0.3 g, MgSO_4_·7H_2_O 0.5 g, FeSO_4_·7H_2_O 0.01 g, ZnSO_4_·7H_2_O 0.001 g, MnCl_2_·2H_2_O 0.001 g, agar 18.0 g, H_2_O 1000 mL, pH 7.4-7.6 |
|  | SCA (starch casein agar) | Soluble starch 10.0 g, casein 0.3 g, KNO_3_ 2.0 g, NaCl 2.0 g, K_2_HPO_4_ 2.0 g, MgSO_4_·7H_2_O 0.05 g, CaCO_3_ 0.02 g, FeSO_4_·7H_2_O 0.01 g, Vitamins (0.5 mg each of thiamine-HCl, riboflavin, niacin, pyridoxin-HCl, inositol, Ca-pantothenat, p-aminobenzoic acid, and 0.25 mg of biotin), agar 18.0 g, H_2_O 1000 mL, pH 7.5 |
|  | GYM (GYM *Streptomyces* Agar) | Malt extract 10.0 g, yeast extract 4.0 g, glucose 4 g, CaCO_3_ 2.0 g, agar 18.0 g, H_2_O 1000 mL, pH 7.2 |
|  | GS (Gause’s No. 1) | Soluble starch 20 g, KNO_3_ 1.0 g, K_2_HPO_4_·3H_2_O 0.5 g, MgSO_4_·7H_2_O 0.5 g, NaCl 0.5 g, FeSO_4_·7H_2_O 0.01 g, agar 18 g, H_2_O 1000 mL, pH 7.4-7.6 |
|  | M-HV (modified HV medium) | Soluble starch 2.0 g, KNO_3_ 0.5 g, KCl 1.71 g, Na_2_HPO_4_ 0.5 g, CaCO_3_ 0.02 g, MgSO_4_·7H_2_O 0.05 g, FeSO_4_·7H_2_O 0.01 g, HV Multi-Vitamins (thiamine 0.05 g, riboflavin 0.05 g, inose 0.05 g, pantothenic acid 0.05 g, p-aminobenzoic acid 0.05 g, vitamin B6 0.05 g, biotin 0.025 g, niacin 0.05 g, H_2_O 100 mL), agar 18.0 g, H_2_O 1000 mL |
| Cultivation | GS (Gause’s No. 1) | Soluble starch 20 g, KNO_3_ 1.0 g, K_2_HPO_4_·3H_2_O 0.5 g, MgSO_4_·7H_2_O 0.5 g, NaCl 0.5 g, FeSO_4_·7H_2_O 0.01 g, agar 18 g, H_2_O 1000 mL, pH 7.4-7.6 |
|  | LB (Luria Bertani) | Yeast extract 5.0 g, NaCl 10.0 g, peptone 10.0 g, agar 18.0 g, H_2_O 1000 mL |

**Table S2.** 16S rRNA similarity values of cultivable actinomycetes isolates with closely related species

| **Strains** | **Source** | **Closest Type Strain** | **Accession no.** | **Similarity (%)** | **GenBank accession no.** |
| --- | --- | --- | --- | --- | --- |
| ZLB-11 | nymph cuticle | *Streptomyces cavourensis* | NBRC 13026 | 99.71 | PP456360 |
| ZLB-12 | nymph cuticle | *Streptomyces cavourensis* | NBRC 13026 | 99.64 | PP456365 |
| ZLB-13 | nymph cuticle | *Streptomyces cavourensis* | NBRC 13026 | 99.71 | PP456358 |
| ZLB-14 | nymph cuticle | *Streptomyces pratensis* | ch24 | 99.27 | PP456317 |
| ZLB-15 | nymph cuticle | *Streptomyces cavourensis* | NBRC 13026 | 99.86 | PP456361 |
| ZLB-16 | nymph cuticle | *Streptomyces pratensis* | ch24 | 99.27 | PP456304 |
| ZLB-17 | nymph cuticle | *Streptomyces pratensis* | ch24 | 99.27 | PP456298 |
| ZLB-18 | nymph cuticle | *Streptomyces pratensis* | ch24 | 99.27 | PP456299 |
| ZLB-20 | nymph cuticle | *Streptomyces albidoflavus* | DSM 40455 | 99.08 | PP456295 |
| ZLB-21 | adults cuticle | *Streptomyces pratensis* | ch24 | 99.27 | PP456359 |
| ZLB-22 | adults cuticle | *Streptomyces cavourensis* | NBRC 13026 | 99.57 | PP456333 |
| ZLB-23 | adults cuticle | *Streptomyces cavourensis* | NBRC 13026 | 99.78 | PP456362 |
| ZLB-24 | adults cuticle | *Streptomyces cavourensis* | NBRC 13026 | 99.86 | PP456363 |
| ZLB-25 | adults cuticle | *Streptomyces pratensis* | ch24 | 99.27 | PP456320 |
| ZLB-26 | adults cuticle | *Streptomyces cavourensis* | NBRC 13026 | 99.36 | PP456296 |
| ZLB-27 | adults cuticle | *Streptomyces pratensis* | ch24 | 99.27 | PP456318 |
| ZLB-29 | adults cuticle | *Streptomyces cavourensis* | NBRC 13026 | 99.78 | PP456364 |
| ZLB-30 | adults cuticle | *Streptomyces pratensis* | ch24 | 99.71 | PP456351 |
| ZLB-32 | adults cuticle | *Streptomyces pratensis* | ch24 | 99.93 | PP456297 |
| ZLB-39 | adults cuticle | *Streptomyces asenjonii* | KNN 35.1b | 98.48 | PP456344 |
| ZLB-43 | adults cuticle | *Streptomyces pratensis* | ch24 | 99.78 | PP456350 |
| ZLC-10 | nymph gut | *Streptomyces pratensis* | ch24 | 99.86 | PP456301 |
| ZLC-19 | nymph gut | *Streptomyces cavourensis* | NBRC 13026 | 99.86 | PP456332 |
| ZLC-20 | nymph gut | *Streptomyces araujoniae* | ASBV-1 | 99.36 | PP456355 |
| ZLC-21 | nymph gut | *Streptomyces daghestanicus* | NRRL B-5418 | 99.08 | PP456353 |
| ZLC-22 | nymph gut | *Streptomyces tendae* | ATCC 19812 | 99.93 | PP456328 |
| ZLC-24 | nymph gut | *Streptomyces violascens* | ISP 5183 | 99.14 | PP456357 |
| ZLC-25 | nymph gut | *Streptomyces albidoflavus* | DSM 40455 | 99.28 | PP456329 |
| ZLC-26 | nymph gut | *Streptomyces violascens* | ISP 5183 | 99.21 | PP456294 |
| ZLC-27 | nymph gut | *Streptomyces albidoflavus* | DSM 40455 | 99.57 | PP456349 |
| ZLC-29 | nymph gut | *Streptomyces intermedius* | NBRC 13049 | 99.16 | PP456289 |
| ZLC-31 | nymph gut | *Streptomyces violascens* | ISP 5183 | 99.14 | PP456354 |
| ZLC-32 | nymph gut | *Streptomyces intermedius* | NBRC 13049 | 99.1 | PP456290 |
| ZLC-33 | nymph gut | *Streptomyces albidoflavus* | DSM 40455 | 99.1 | PP456285 |
| ZLC-34 | nymph gut | *Streptomyces violascens* | ISP 5183 | 98.15 | PP456356 |
| ZLC-36 | nymph gut | *Streptomyces rochei* | NRRL B-2410 | 99.78 | PP456327 |
| ZLC-37 | nymph gut | *Streptomyces daghestanicus* | NRRL B-5418 | 99.5 | PP456330 |
| ZLC-38 | nymph gut | *Streptomyces intermedius* | NBRC 13049 | 99.17 | PP456288 |
| ZLC-39 | nymph gut | *Streptomyces violascens* | ISP 5183 | 99.36 | PP456293 |
| ZLC-40 | nymph gut | *Streptomyces violascens* | ISP 5183 | 98.44 | PP456305 |
| ZLC-41 | nymph gut | *Streptomyces violascens* | ISP 5183 | 99.64 | PP456352 |
| ZLC-42 | nymph gut | *Streptomyces albidoflavus* | DSM 40455 | 99.51 | PP456287 |
| ZLC-43 | nymph gut | *Streptomyces albidoflavus* | DSM 40455 | 99.17 | PP456280 |
| ZLC-44 | nymph gut | *Streptomyces intermedius* | NBRC 13049 | 99.21 | PP456326 |
| ZLC-45 | nymph gut | *Streptomyces pratensis* | ch24 | 99.93 | PP456292 |
| ZLC-46 | nymph gut | *Streptomyces badius* | NRRL B-2567 | 100 | PP456286 |
| ZLC-47 | nymph gut | *Streptomyces pratensis* | ch24 | 99.93 | PP456321 |
| ZLC-48 | nymph gut | *Streptomyces pratensis* | ch24 | 99.71 | PP456341 |
| ZLC-49 | nymph gut | *Streptomyces cavourensis* | NBRC 13026 | 99.52 | PP456284 |
| ZLC-50 | nymph gut | *Streptomyces pratensis* | ch24 | 99.93 | PP456319 |
| ZLC-51 | adults gut | *Streptomyces pratensis* | ch24 | 99.93 | PP456308 |
| ZLC-52 | adults gut | *Streptomyces albidoflavus* | DSM 40455 | 99.36 | PP456340 |
| ZLC-54 | adults gut | *Streptomyces coelicoflavus* | NBRC 15399 | 99.79 | PP456335 |
| ZLC-55 | adults gut | *Streptomyces albidoflavus* | DSM 40455 | 99.5 | PP456339 |
| ZLC-56 | adults gut | *Streptomyces badius* | NRRL B-2567 | 99.86 | PP456343 |
| ZLC-57 | adults gut | *Streptomyces pratensis* | ch24 | 99.71 | PP456338 |
| ZLC-58 | adults gut | *Streptomyces pactum* | NBRC 13433 | 99.3 | PP456307 |
| ZLC-59 | adults gut | *Streptomyces pratensis* | ch24 | 99.93 | PP456337 |
| ZLC-61 | adults gut | *Streptomyces albidoflavus* | DSM 40455 | 99.29 | PP456348 |
| ZLC-63 | adults gut | *Streptomyces pratensis* | ch24 | 99.71 | PP456342 |
| ZLC-64 | adults gut | *Streptomyces albidoflavus* | DSM 40455 | 99.36 | PP456347 |
| ZLC-65 | adults gut | *Streptomyces pratensis* | ch24 | 99.93 | PP456346 |
| ZLC-66 | adults gut | *Streptomyces badius* | NRRL B-2567 | 99.79 | PP456345 |
| ZLC-69 | adults gut | *Streptomyces djakartensis* | NBRC 15409 | 98.78 | PP456331 |
| ZLC-71 | adults gut | *Streptomyces pratensis* | ch24 | 99.93 | PP456322 |
| ZLC-72 | adults gut | *Streptomyces cavourensis* | NBRC 13026 | 99.93 | PP456334 |
| ZLC-77 | adults gut | *Streptomyces pratensis* | ch24 | 99.93 | PP456325 |
| ZLC-78 | adults gut | *Streptomyces coelicoflavus* | NBRC 15399 | 100 | PP456336 |
| ZLC-79 | adults gut | *Streptomyces setonii* | NRRL ISP-5322 | 100 | PP456323 |
| ZLC-80 | adults gut | *Streptomyces pratensis* | ch24 | 99.93 | PP456291 |
| ZLC-81 | adults gut | *Streptomyces olivaceus* | NRRL B-3009 | 100 | PP456283 |
| ZLC-85 | adults gut | *Streptomyces olivaceus* | NRRL B-3009 | 99.3 | PP456314 |
| ZLC-86 | adults gut | *Streptomyces violascens* | ISP 5183 | 99.64 | PP456306 |
| ZLC-87 | adults gut | *Nocardiopsis alba* | DSM 43377 | 99.09 | PP456310 |
| ZLC-88 | adults gut | *Streptomyces olivaceus* | NRRL B-3009 | 99.02 | PP456315 |
| ZLC-95 | adults gut | *Streptomyces pratensis* | ch24 | 99.93 | PP456300 |
| ZLC-96 | adults gut | *Streptomyces badius* | NRRL B-2567 | 100 | PP456282 |
| ZLC-97 | adults gut | *Streptomyces badius* | NRRL B-2567 | 100 | PP456281 |
| ZLC-100 | adults gut | *Streptomyces pratensis* | ch24 | 99.93 | PP456303 |
| ZLC-101 | adults gut | *Streptomyces pratensis* | ch24 | 99.93 | PP456312 |
| ZLC-102 | adults gut | *Streptomyces pratensis* | ch24 | 99.93 | PP456316 |
| ZLC-103 | adults gut | *Streptomyces pratensis* | ch24 | 99.93 | PP456313 |
| ZLC-105 | adults gut | *Streptomyces pratensis* | ch24 | 99.93 | PP456309 |
| ZLC-106 | adults gut | *Streptomyces violascens* | ISP 5183 | 99.14 | PP456324 |
| ZLC-107 | adults gut | *Streptomyces pratensis* | ch24 | 99.93 | PP456311 |
| ZLC-112 | adults gut | *Streptomyces olivaceus* | NRRL B-3009 | 99.11 | PP456302 |

**
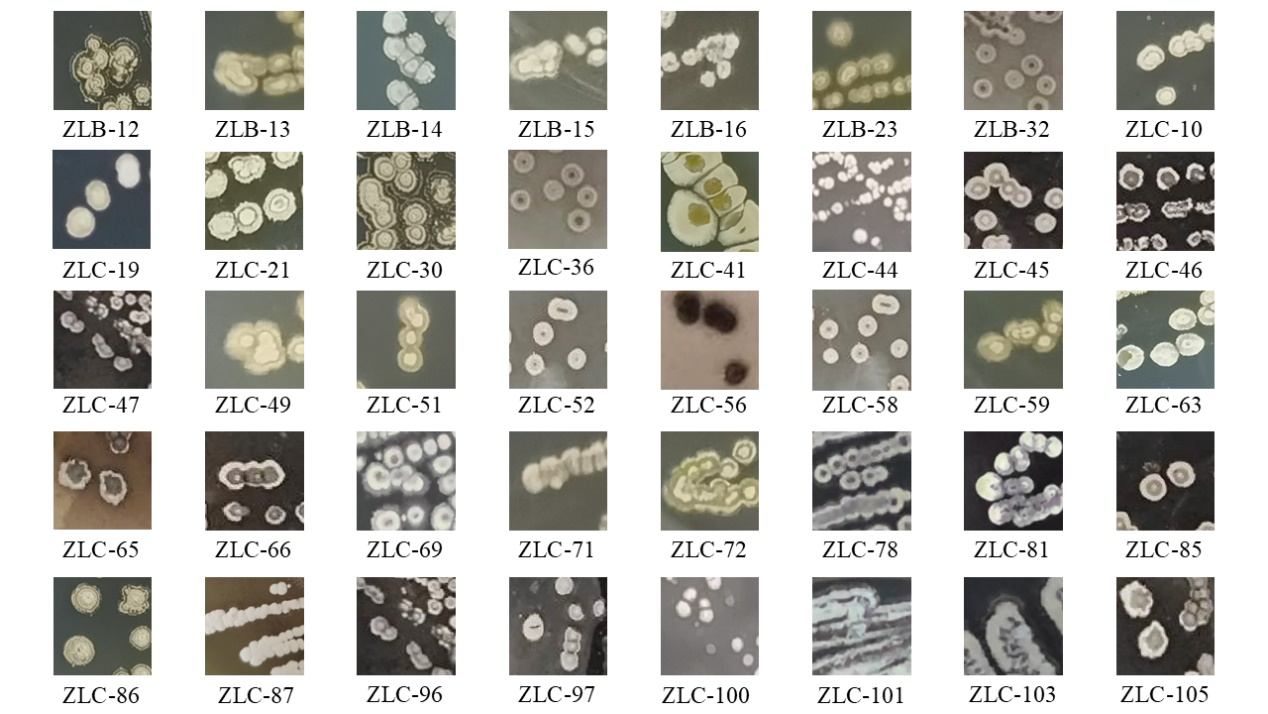
Figure S1.** Colony morphology of part symbiotic actinomycetes.

**
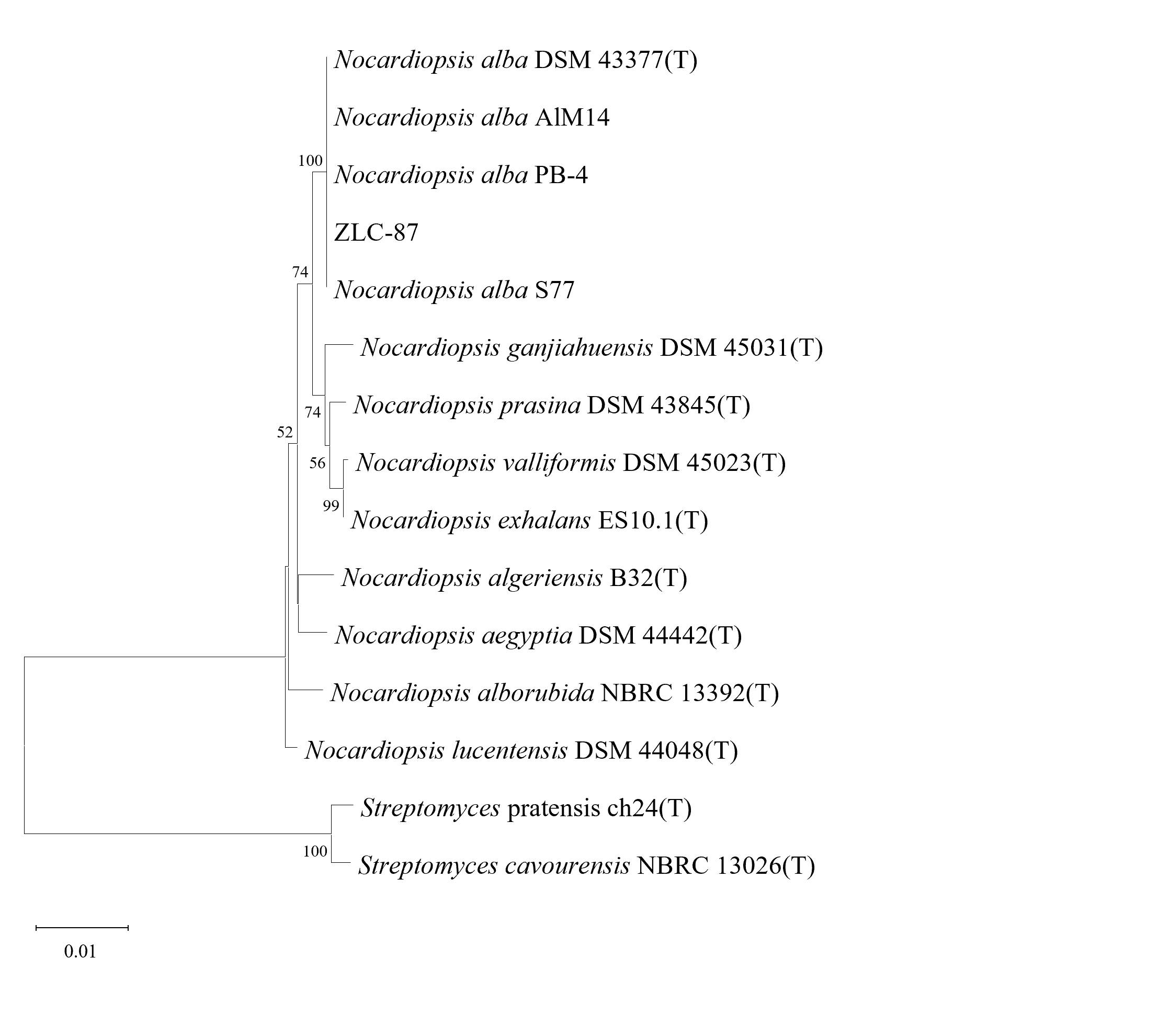
Figure S2.** Neighbor-joining phylogenetic tree of 16S rRNA sequences of ZLC-87

The test of phylogeny was done using bootstrap method with a value of 1000 bootstrap in MEGA 11 software.


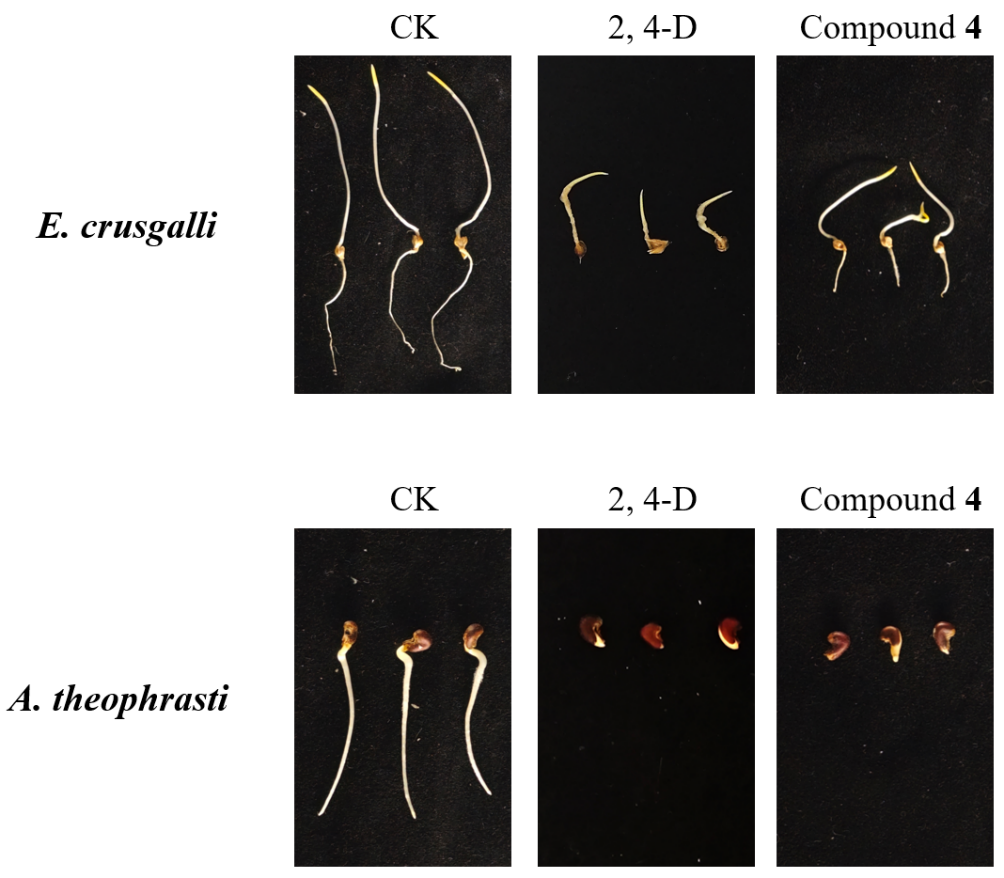
 **Figure S3.** The inhibitory effect of compound **4** on *E. crusgalli* and *A. theophrasti*.


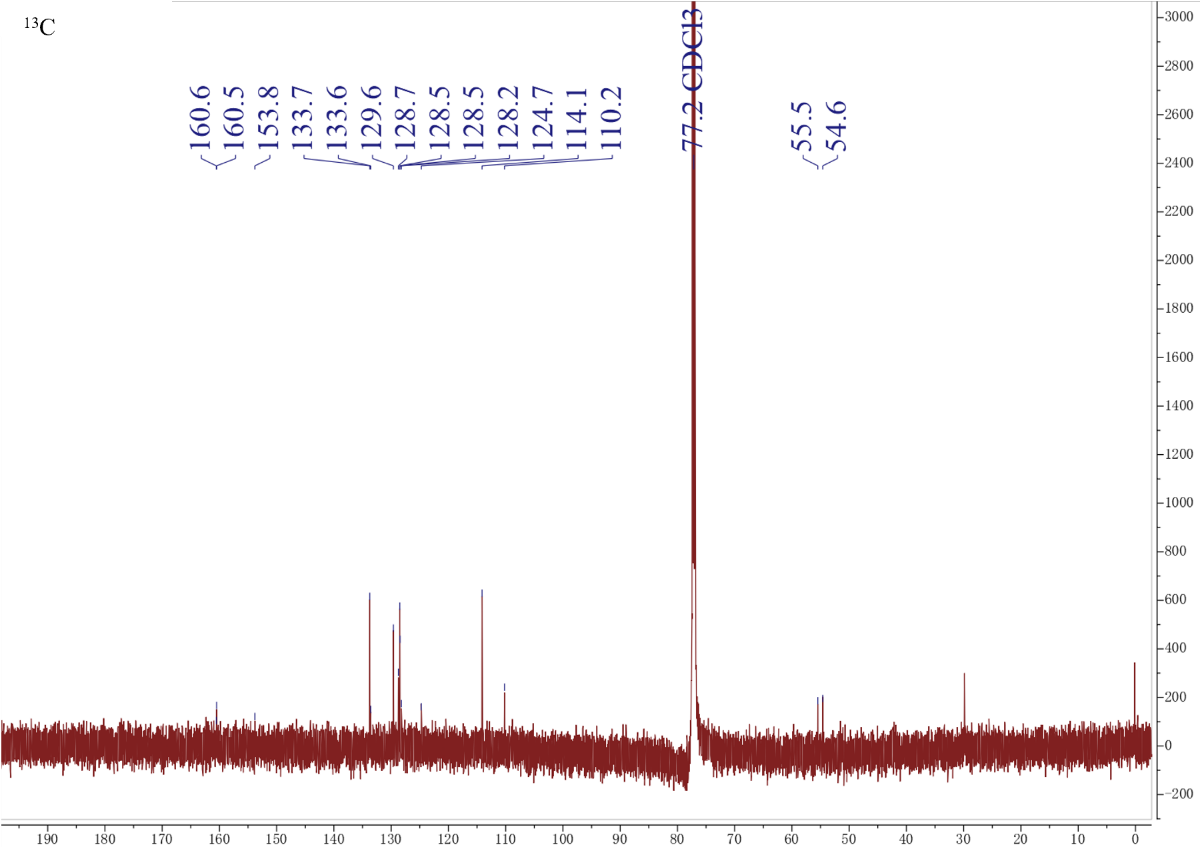

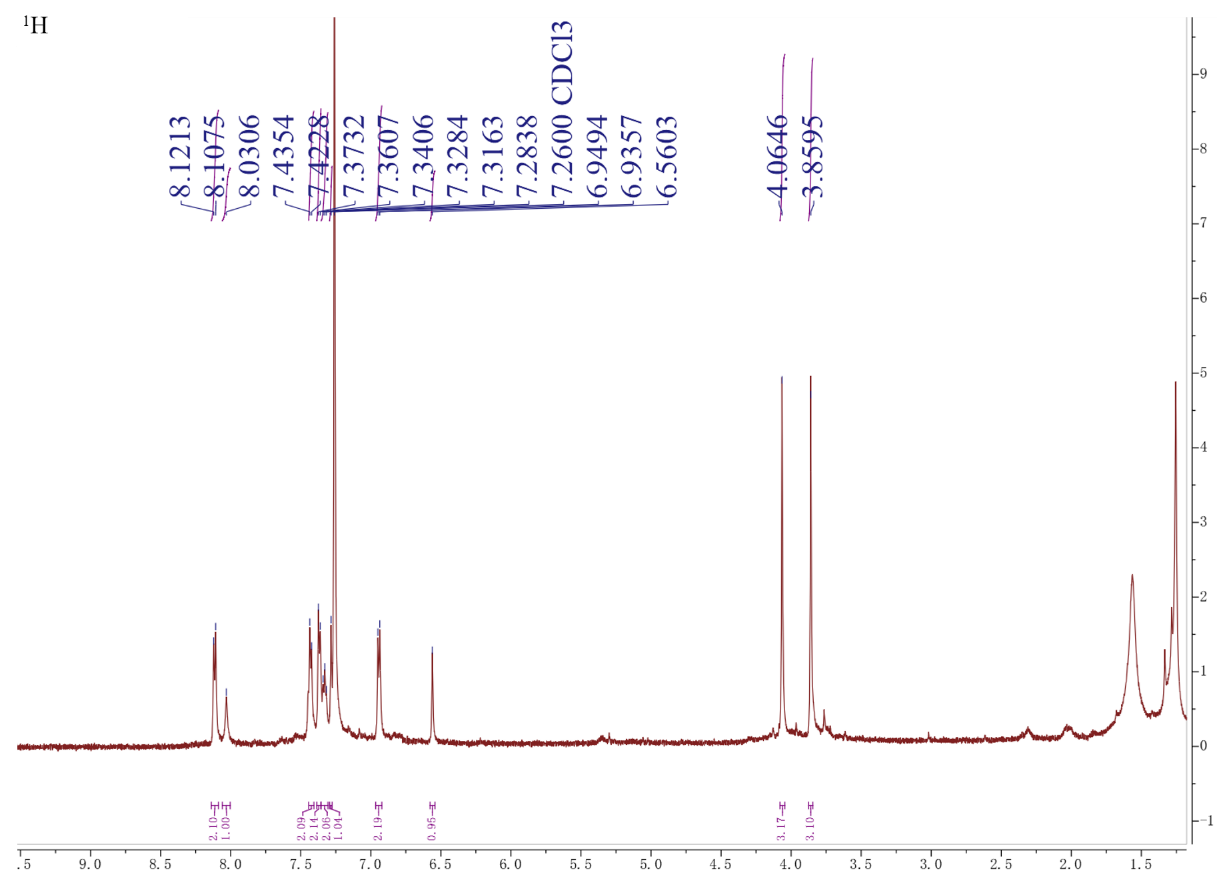
**Figure S4.** The ^1^H NMR spectrum of compound **1** (600 MHz, CDCl_3_).

**Figure S5.** The ^13^C NMR spectrum of compound **1** (150 MHz, CDCl_3_).


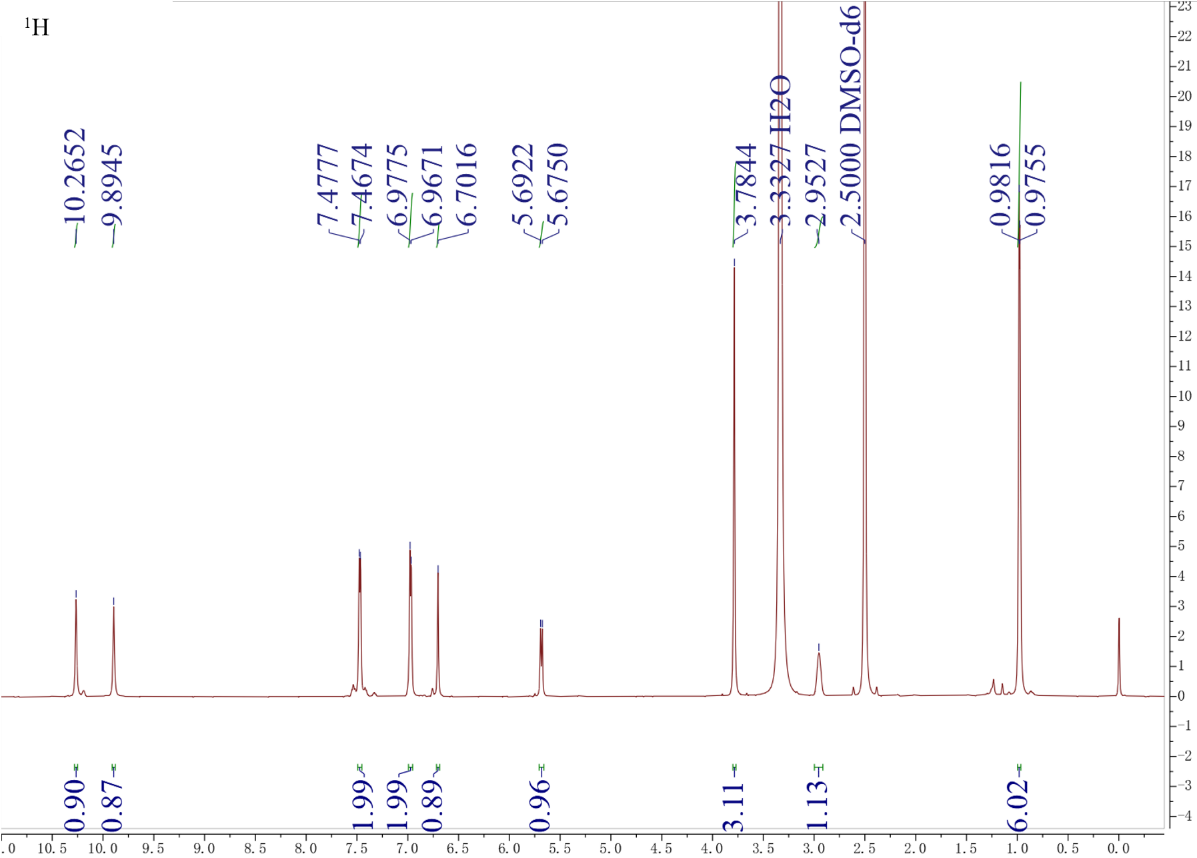

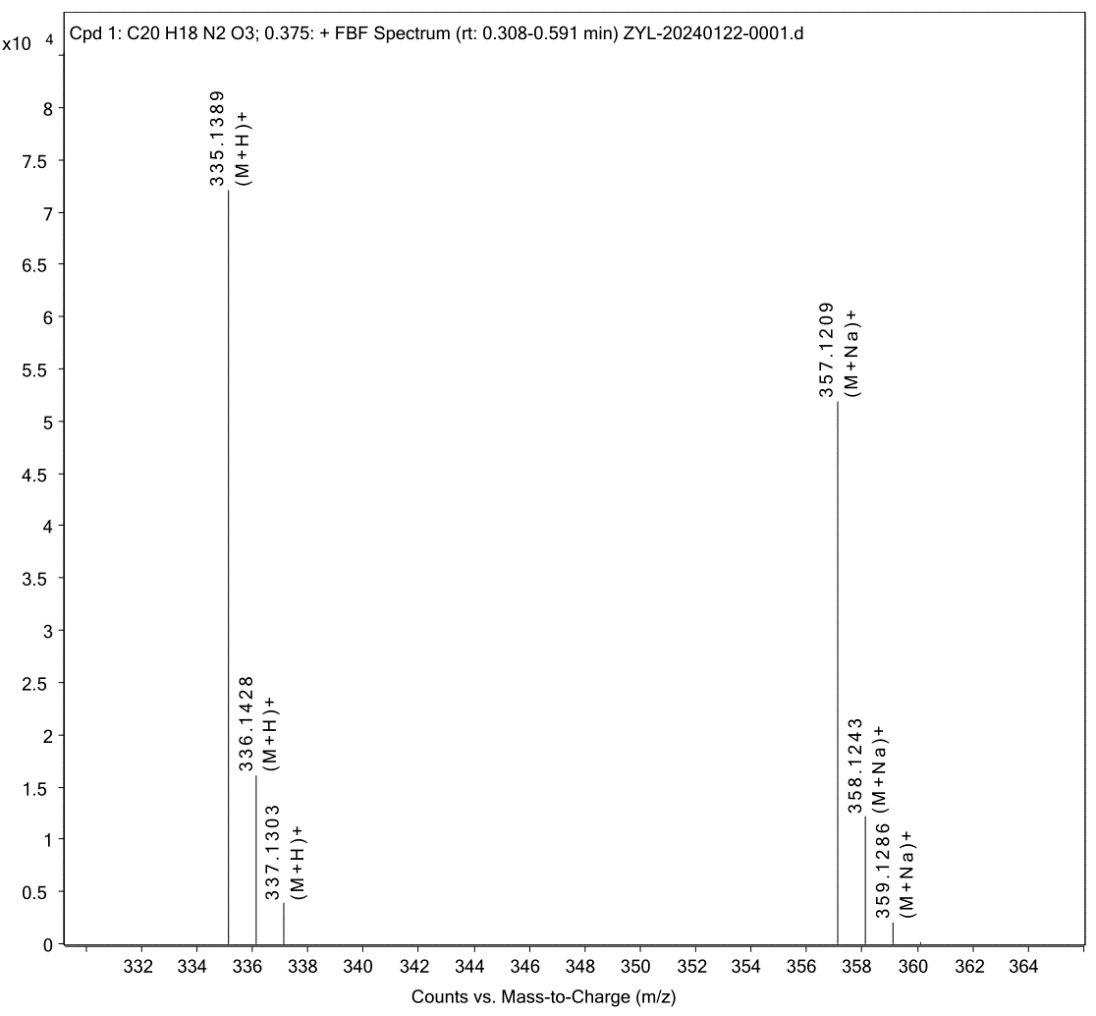
**Figure S6.** The HR-ESI-MS spectrum of compound **1**.

**Figure S7.** The ^1^H NMR spectrum of compound **2** (600 MHz, DMSO- *d_6_*).


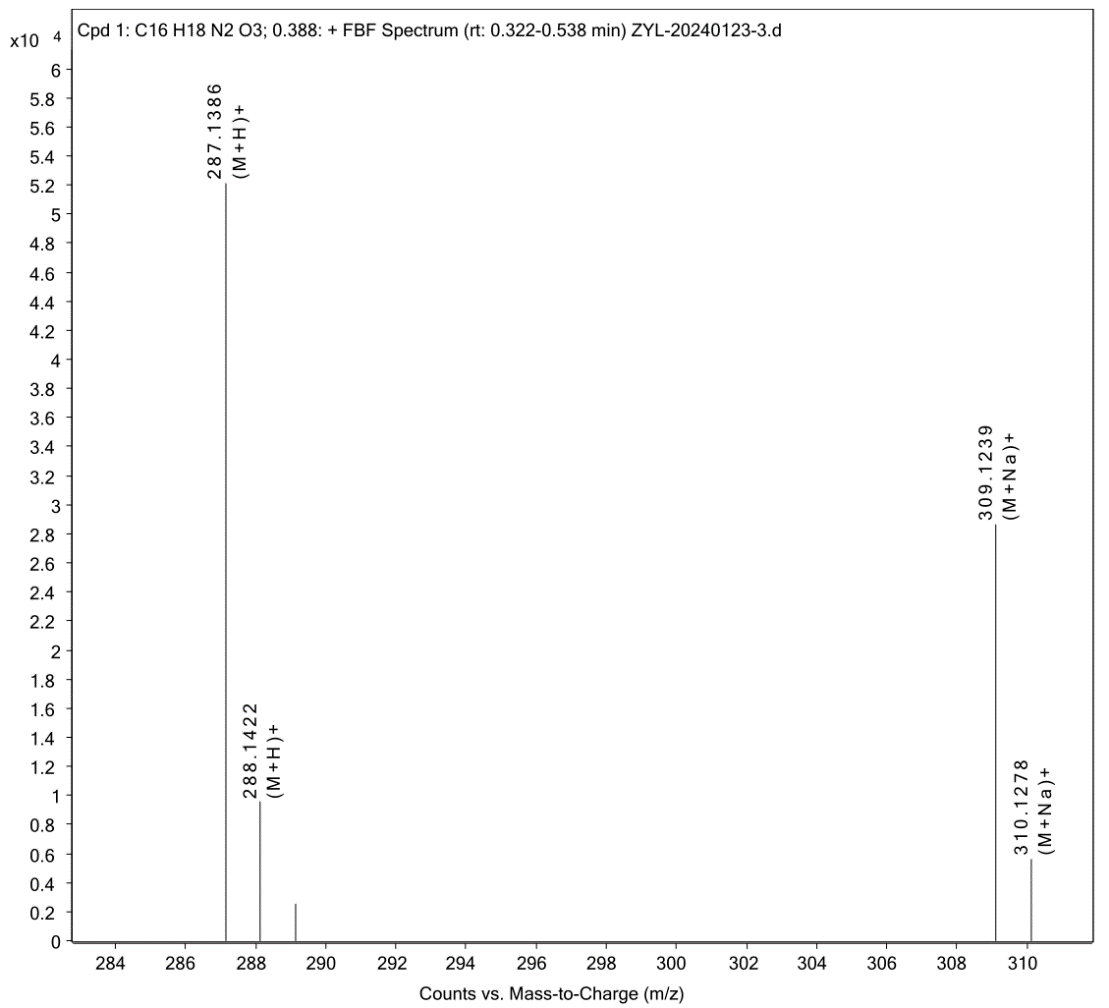

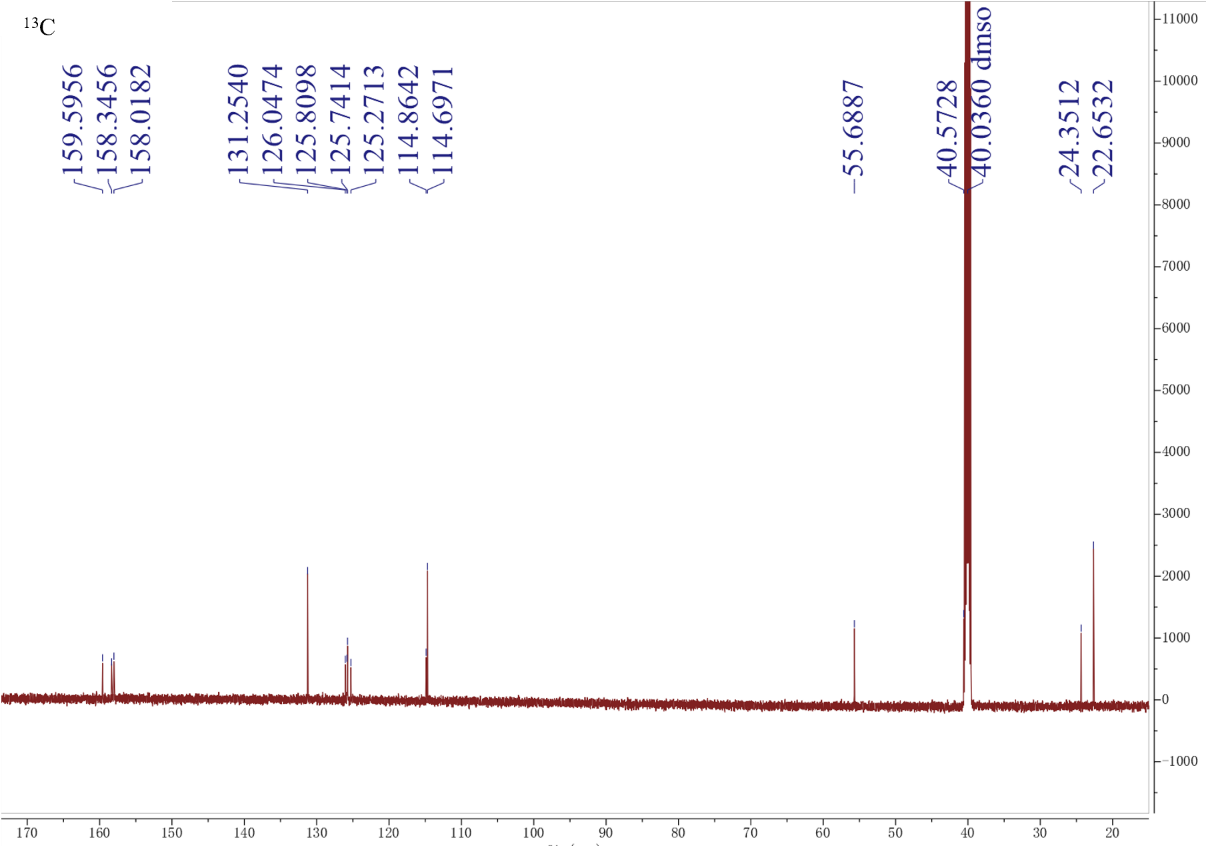
**Figure S8.** The ^13^C NMR spectrum of compound **2** (150 MHz, DMSO- *d_6_*).

**Figure S9.** The HR-ESI-MS spectrum of compound **2**.

**
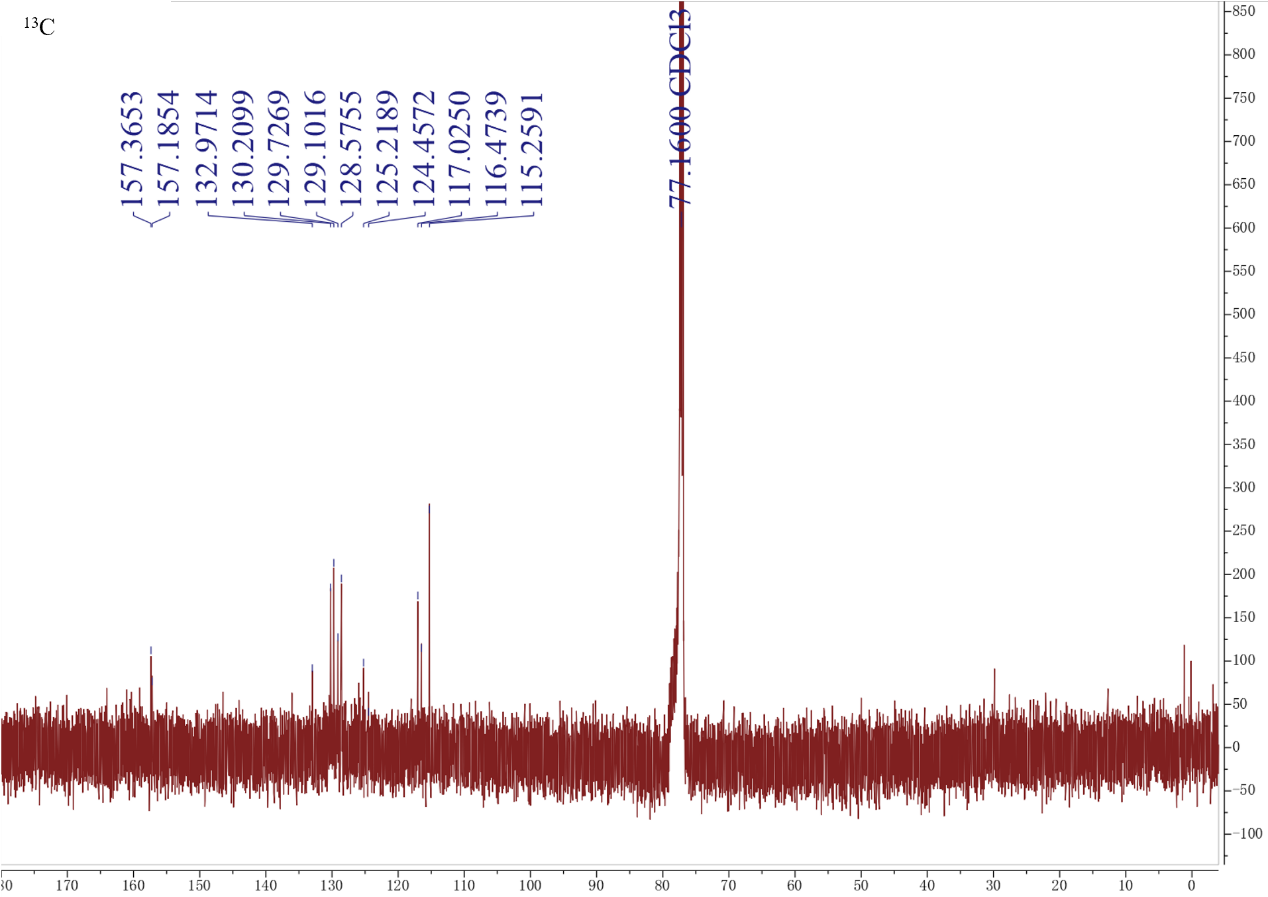
Figure S10.** The ^1^H NMR spectrum of compound **3** (600 MHz, CDCl_3_).
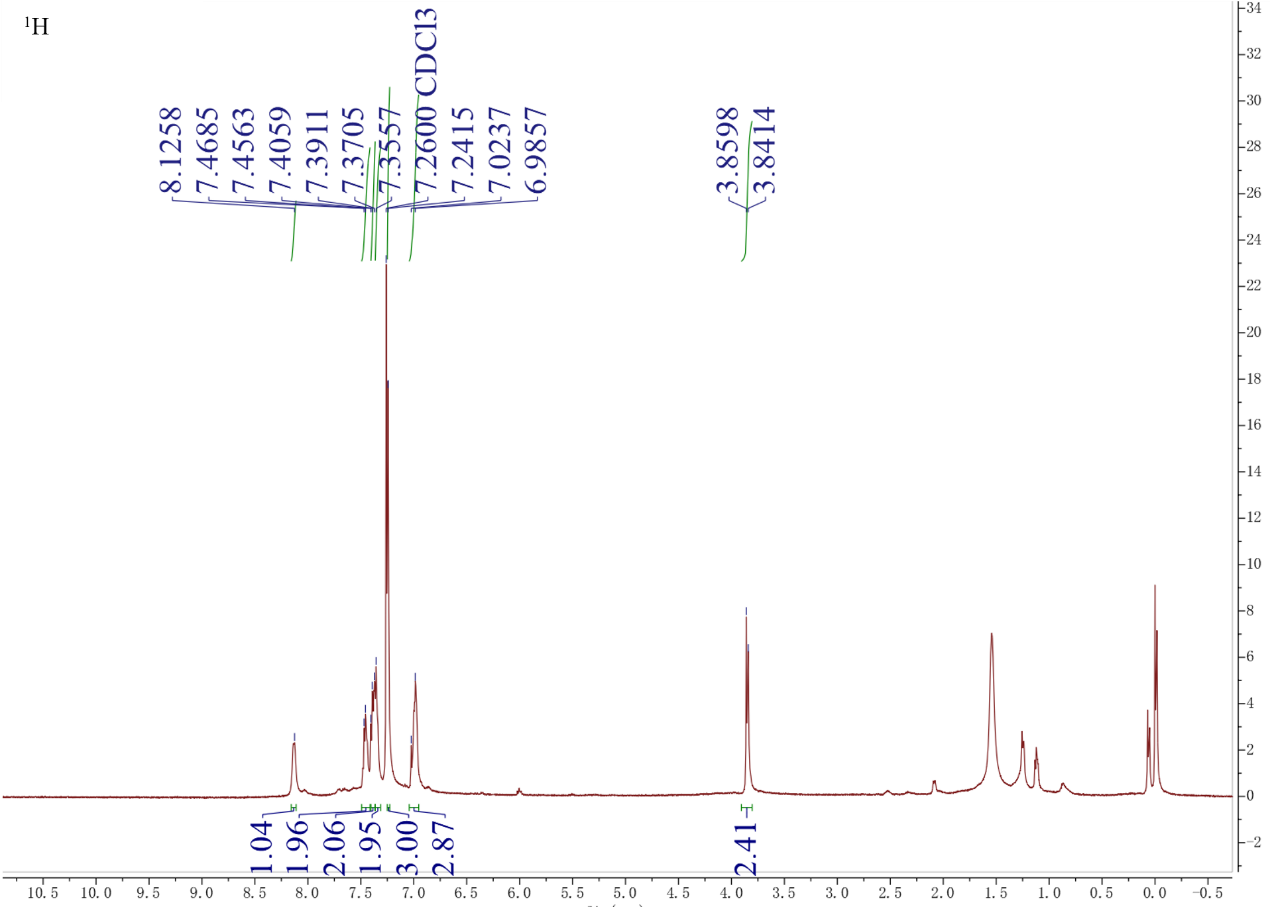


**Figure S11.** The ^13^C NMR spectrum of compound **3** (150 MHz, CDCl_3_).


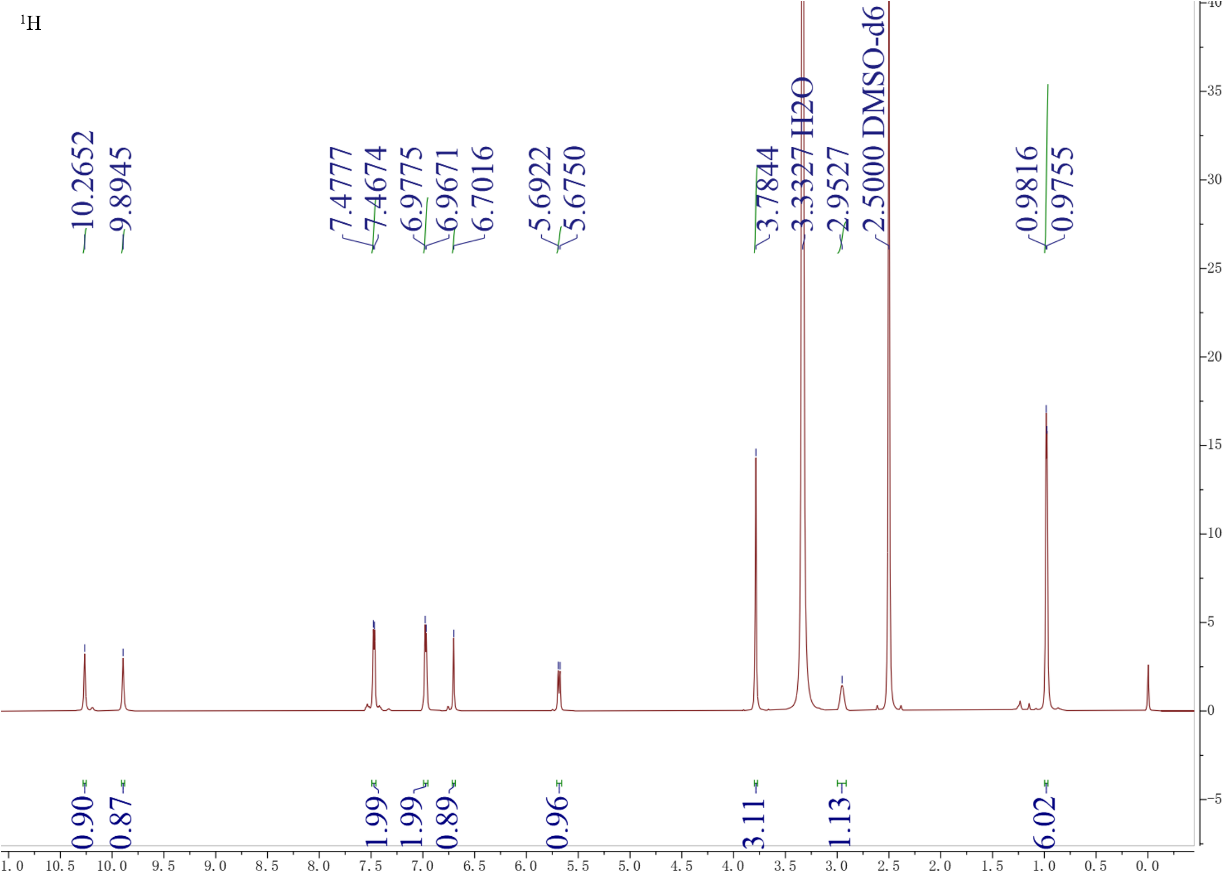
**Figure S12.** The HR-ESI-MS spectrum of compound **3**.
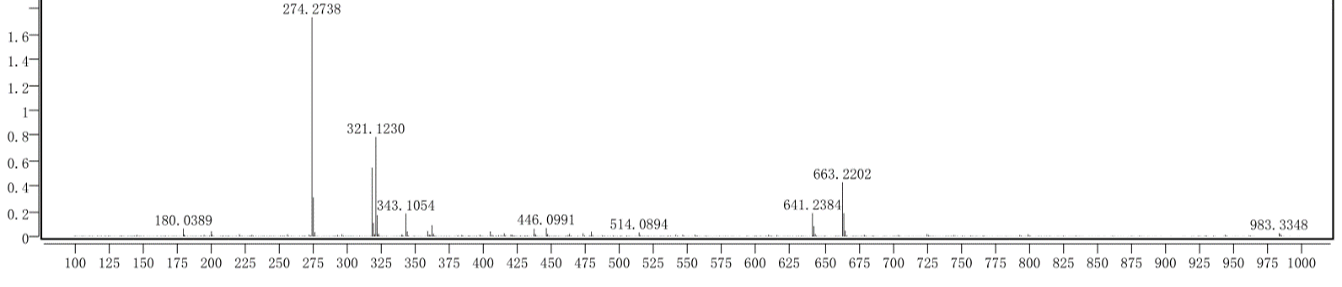


**Figure S13.** The ^1^H NMR spectrum of compound **4** (600 MHz, Acetone-*d_6_*).


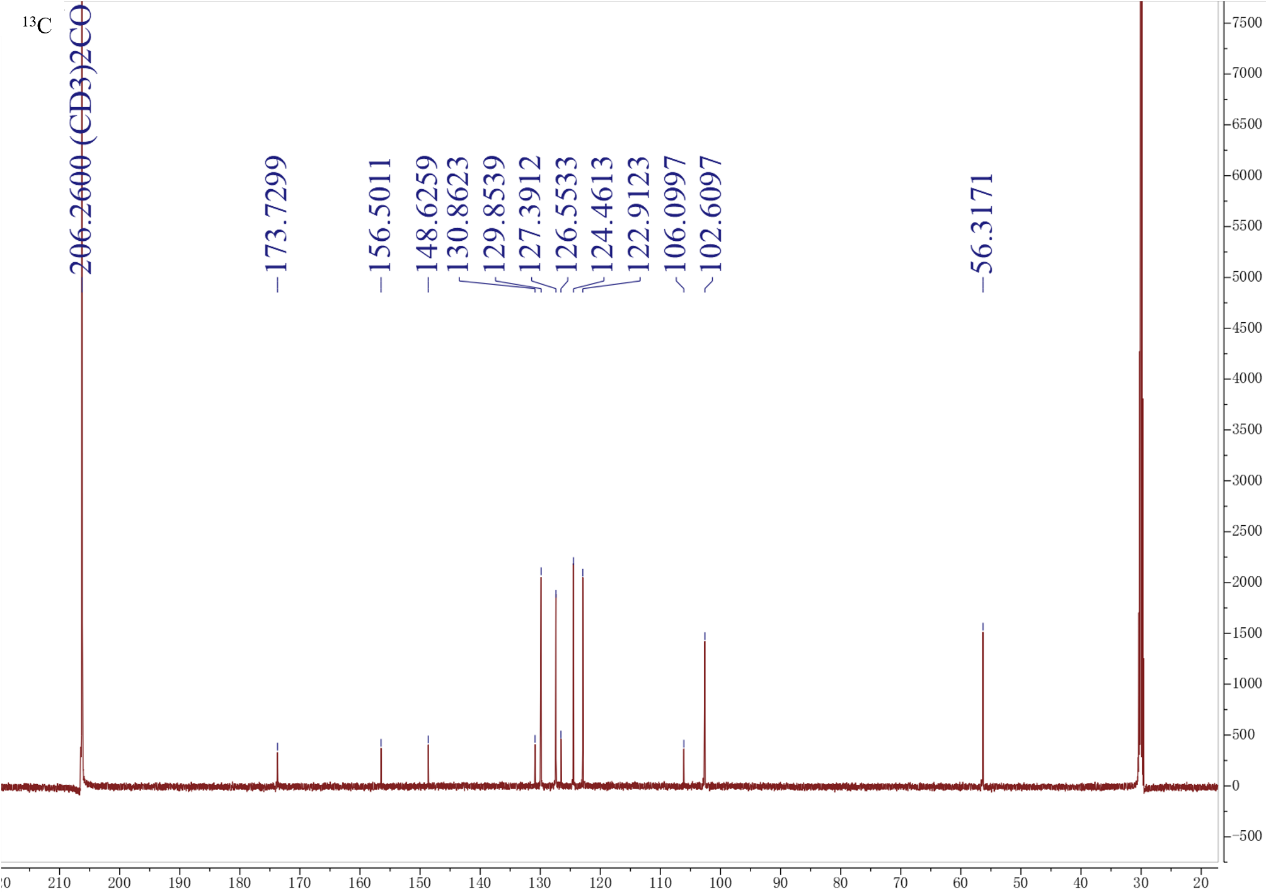
**
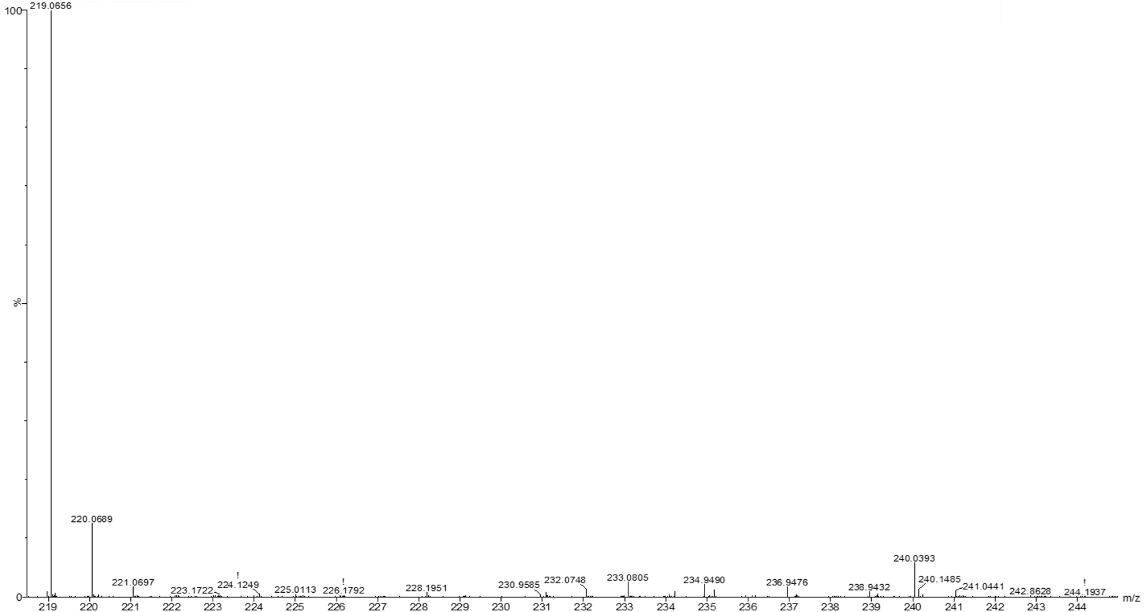
Figure S14.** The ^13^C NMR spectrum of compound **4** (150 MHz, Acetone-*d_6_*).

**Figure S15.** The HR-ESI-MS spectrum of compound **4**.


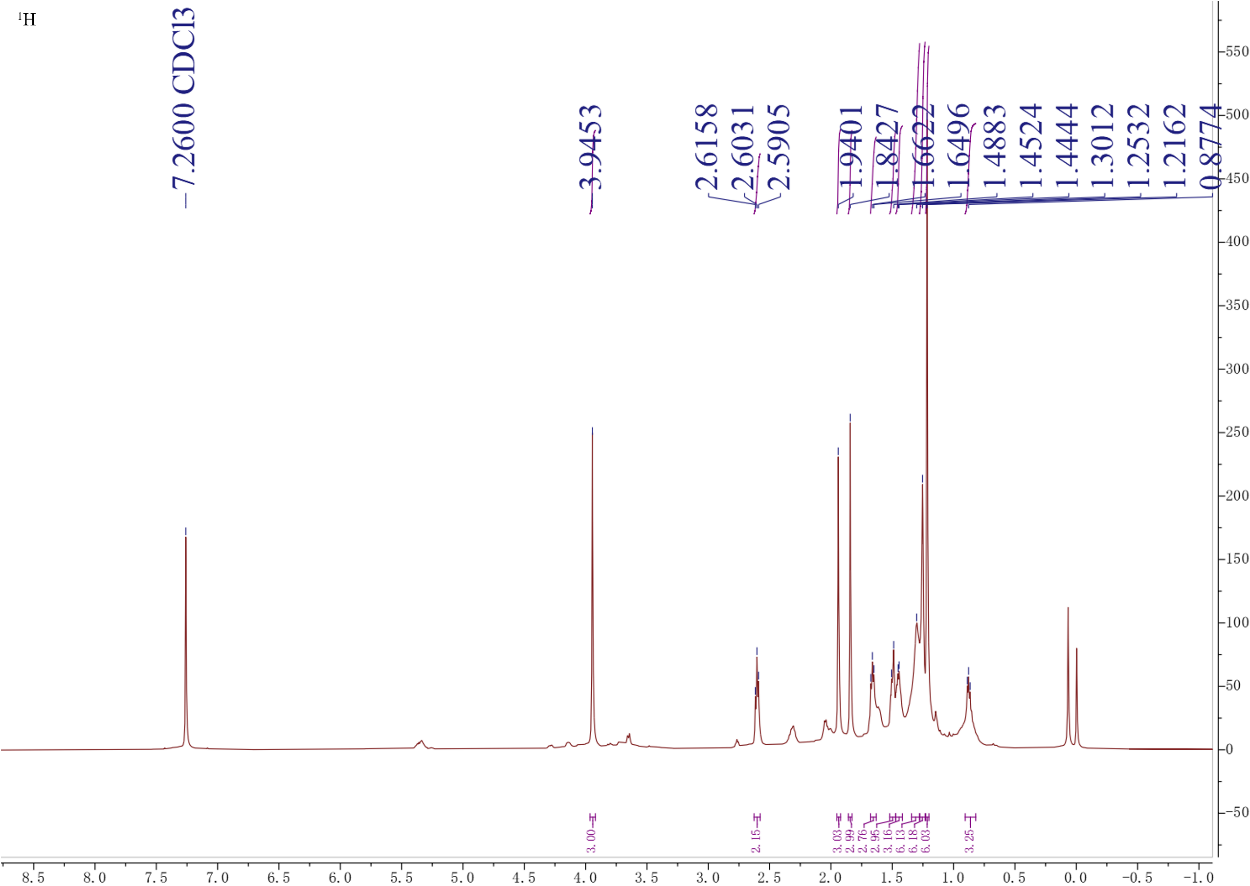


**Figure S16.** The ^1^H NMR spectrum of compound **5** (600 MHz, CDCl_3_).


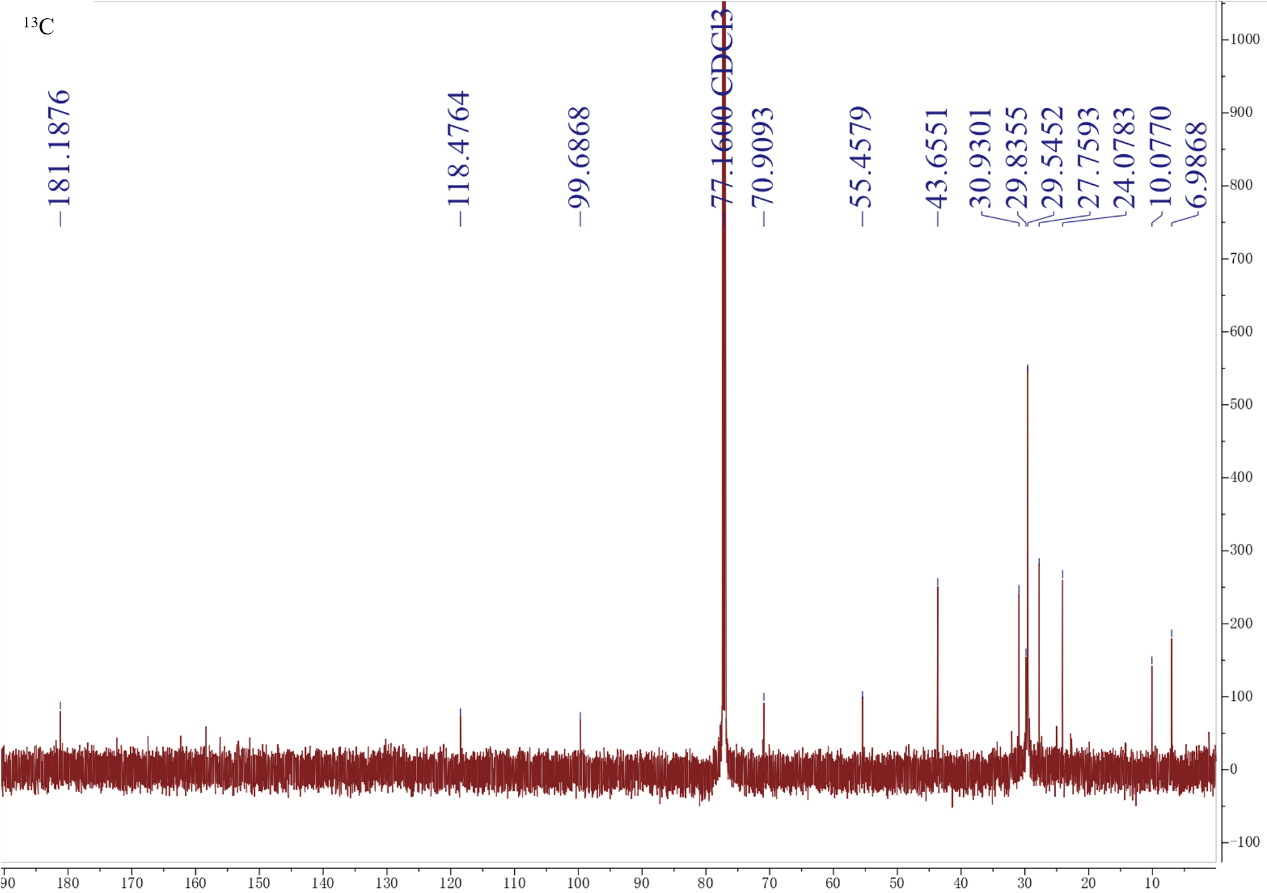


**Figure S17.** The ^13^C NMR spectrum of compound **5** (150 MHz, CDCl_3_).


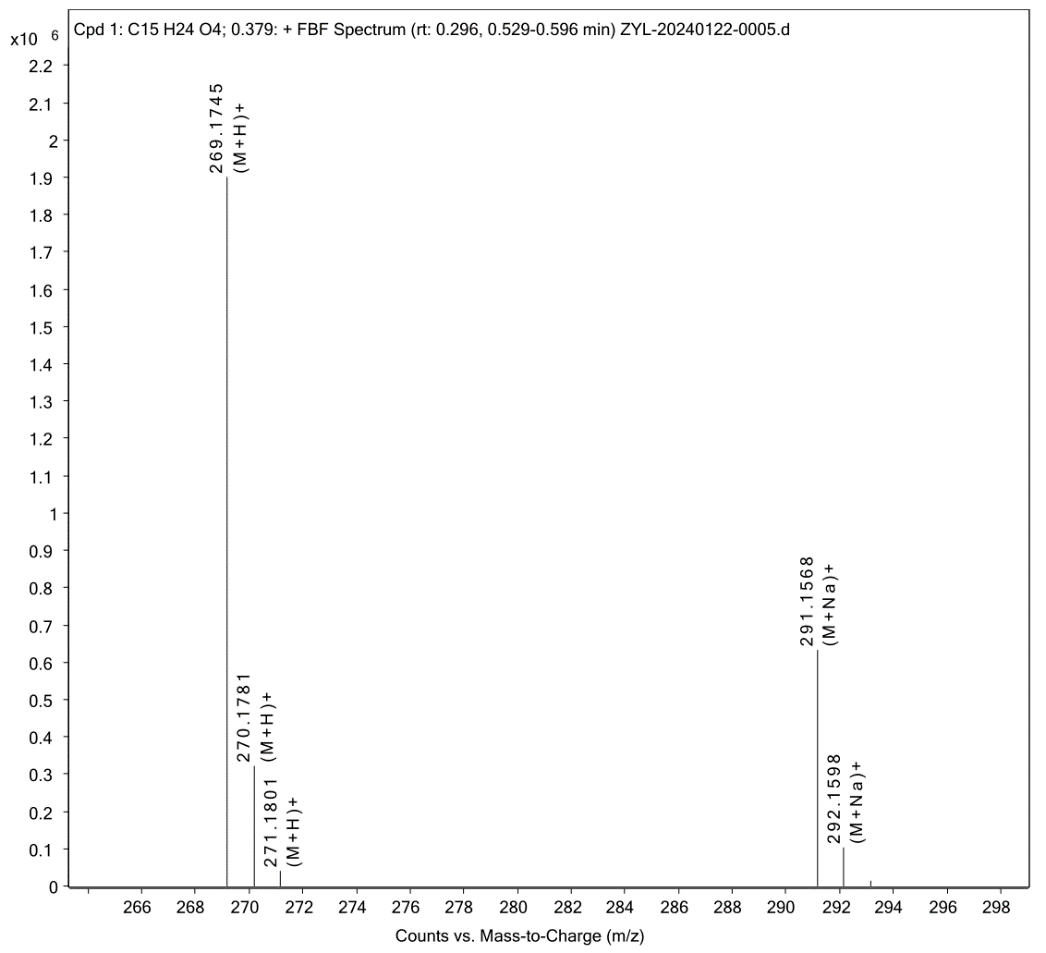
**Figure S18.** The HR-ESI-MS spectrum of compound **5**.


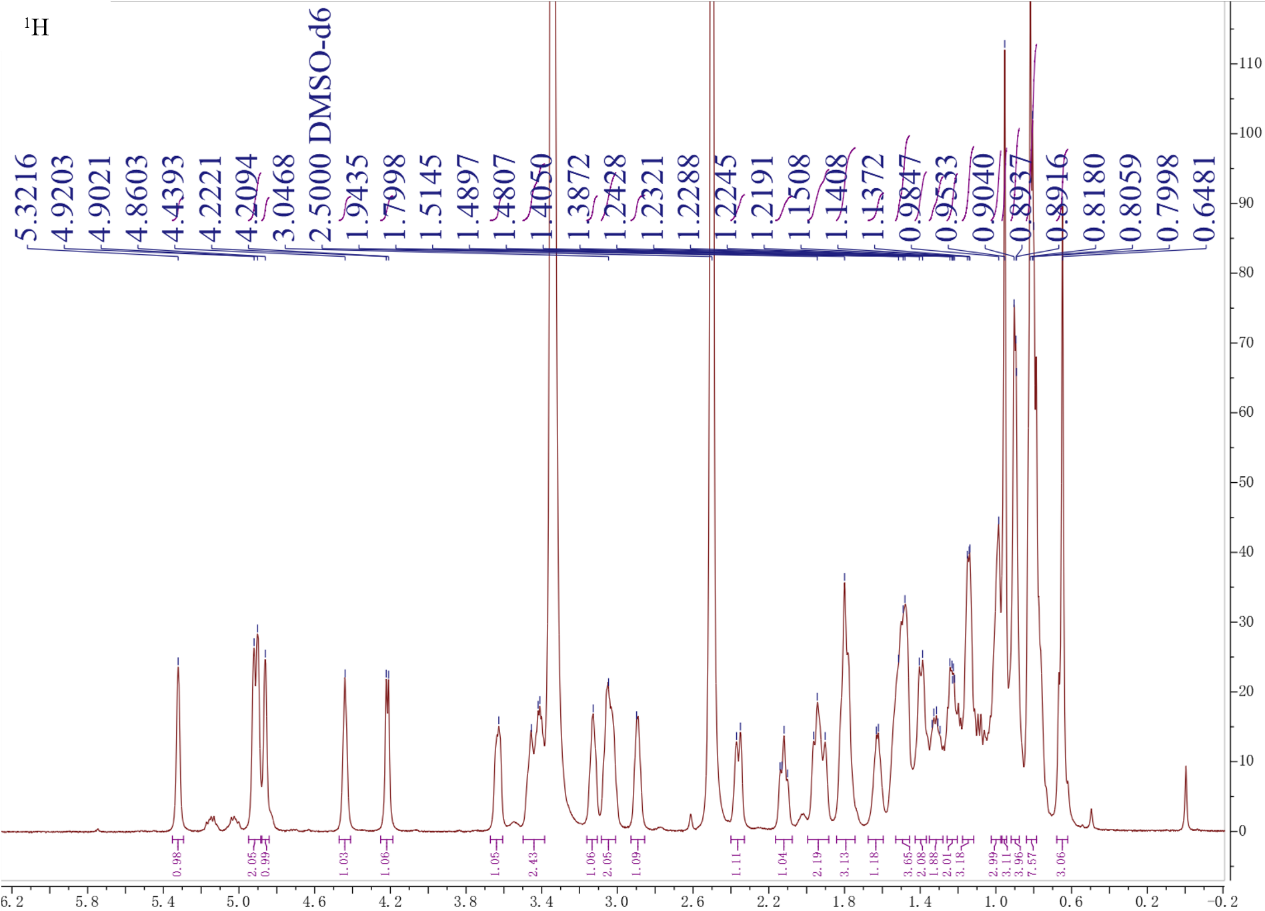


**Figure S19.** The ^1^H NMR spectrum of compound **6** (600 MHz, DMSO- *d_6_*).


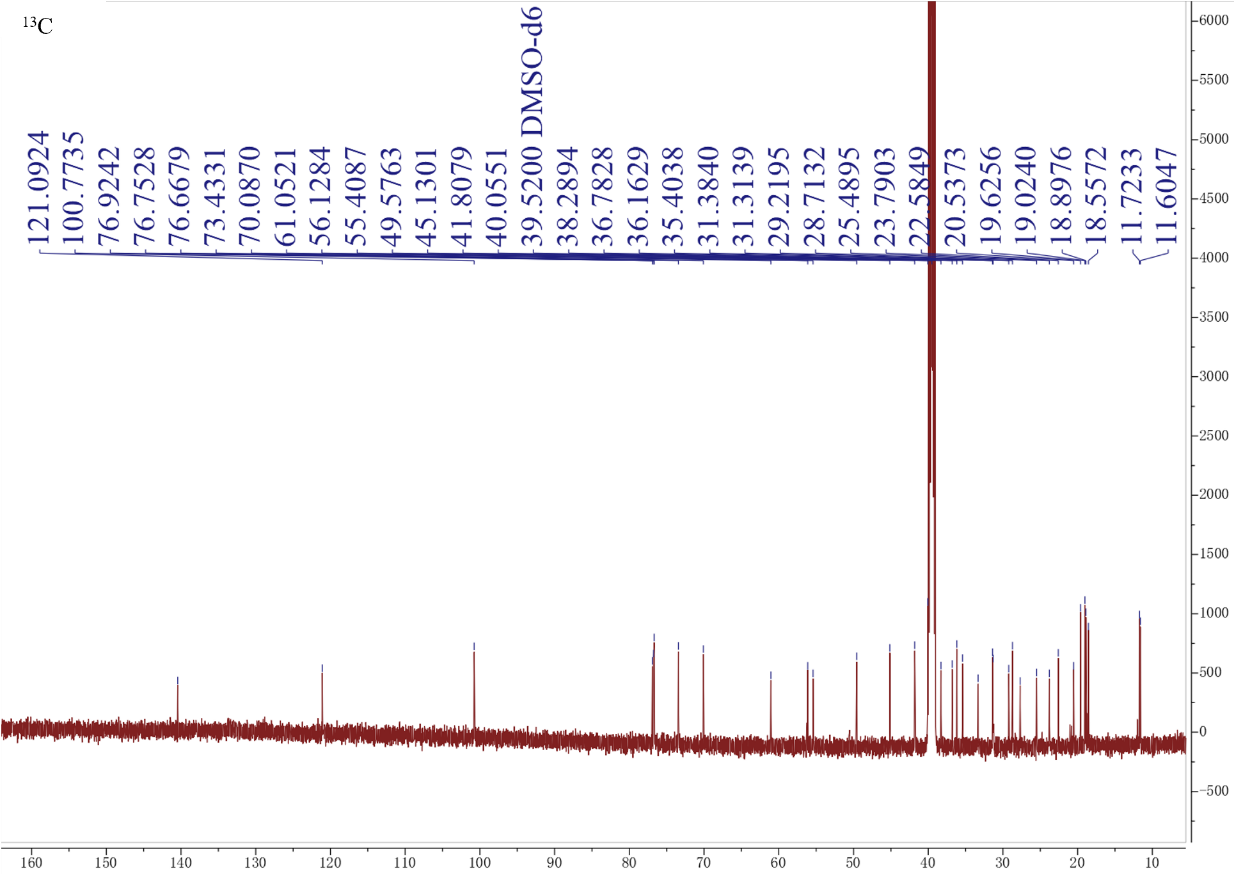


**Figure S20.** The ^13^C NMR spectrum of compound **6** (150 MHz, DMSO- *d_6_*).
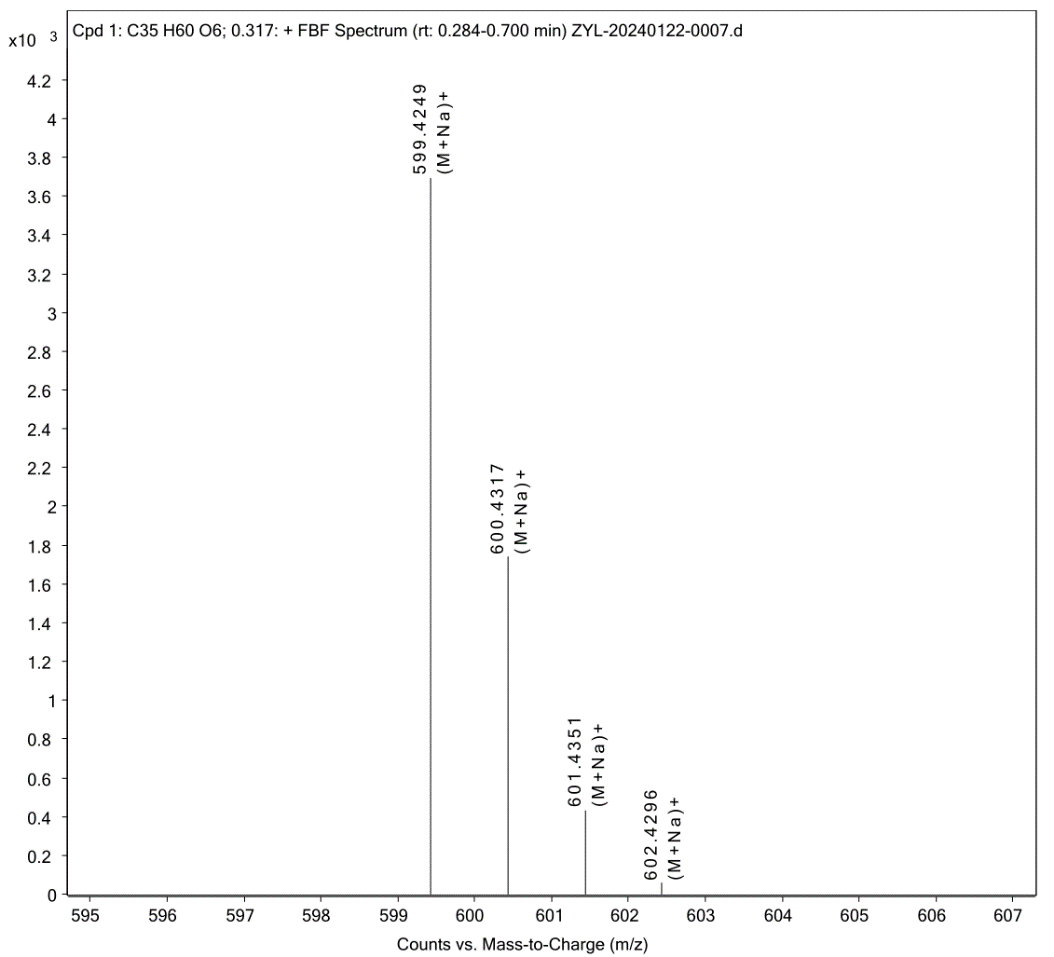


**Figure S21.** The HR-ESI-MS spectrum of compound **6**.


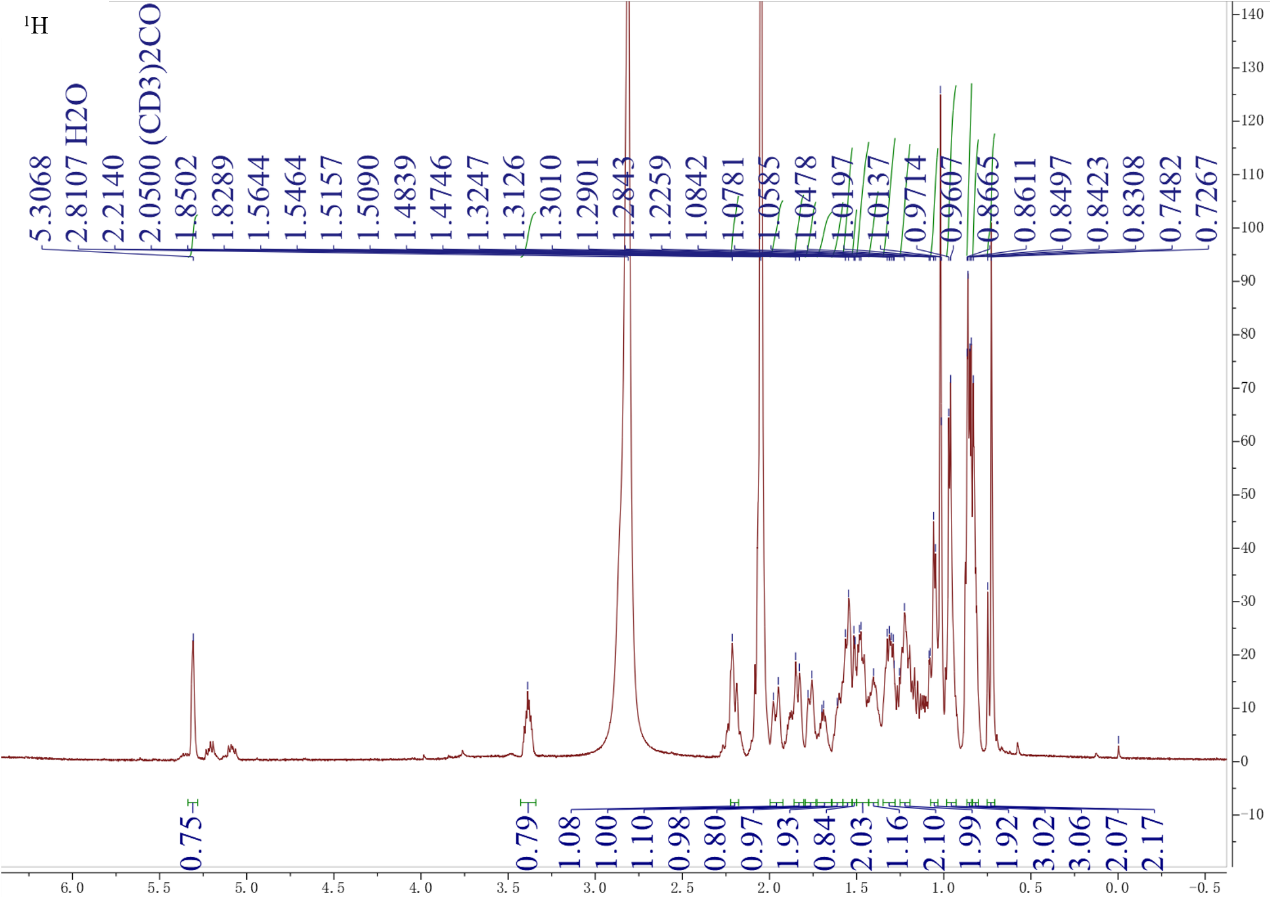


**Figure S22.** The ^1^H NMR spectrum of compound **7** (600 MHz, Acetone-*d_6_*).


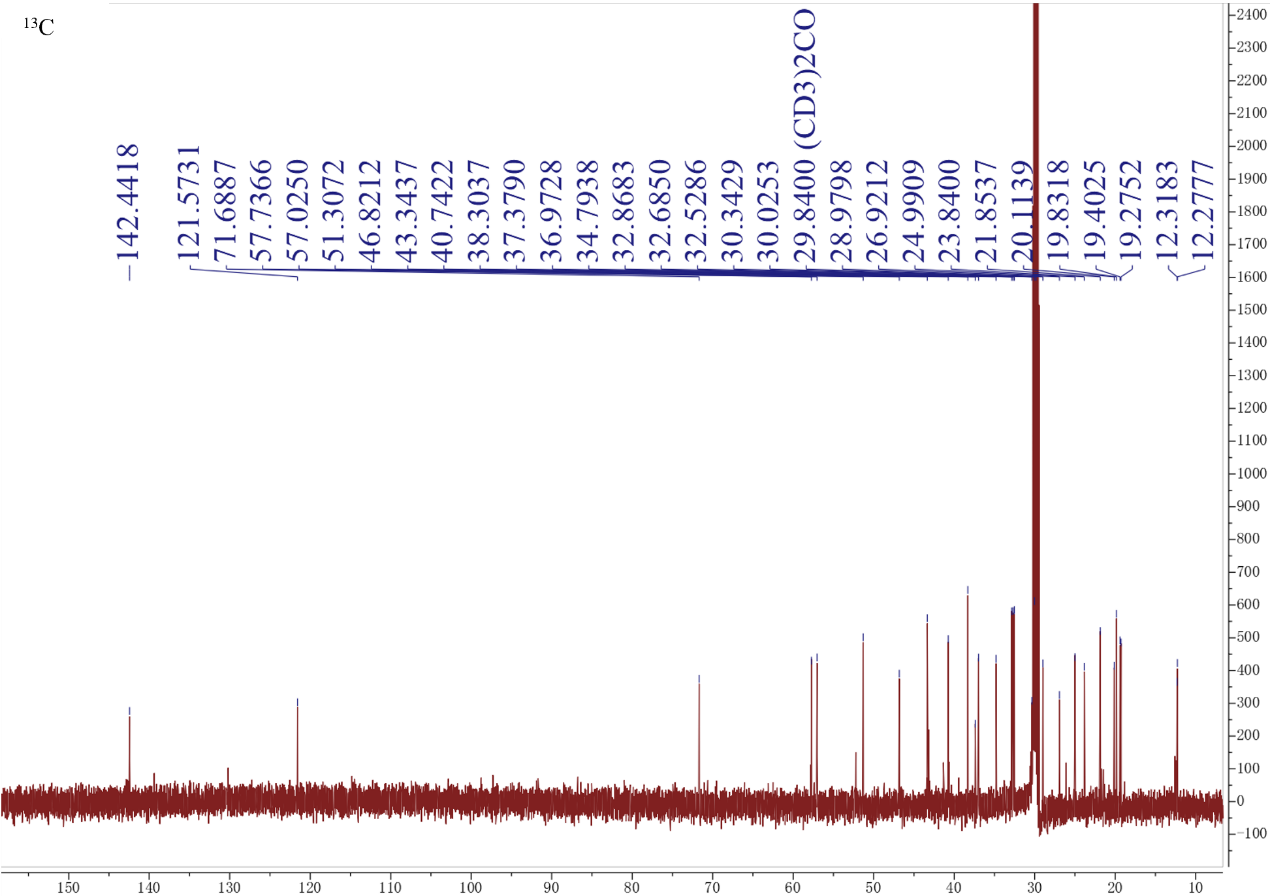


**Figure S23.** The ^13^C NMR spectrum of compound **7** (150 MHz, Acetone-*d_6_*).


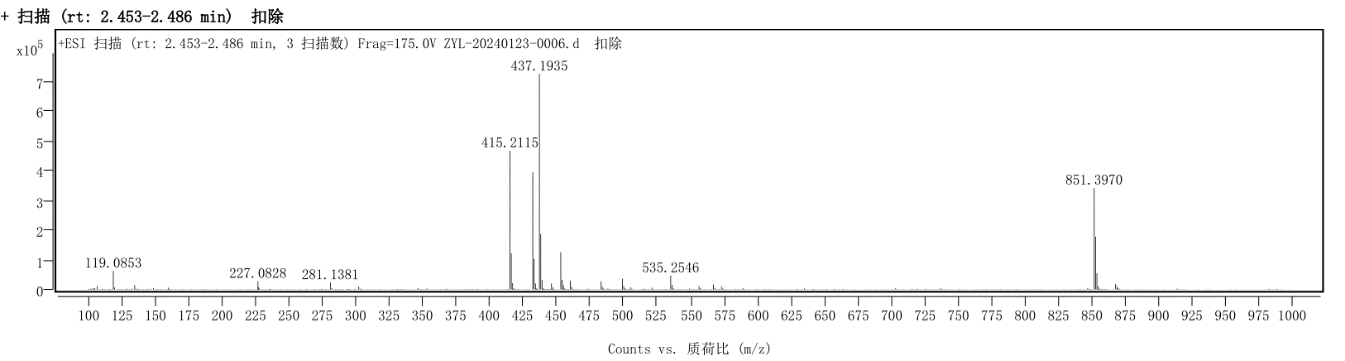


**Figure S24.** The HR-ESI-MS spectrum of compound **7**.
